# Severe Mental Illness: A Contraindication to Watchful Waiting in Hernia Management?

**DOI:** 10.7759/cureus.14915

**Published:** 2021-05-09

**Authors:** David M Milne, Vijay Naraynsingh, Shivan Goolcharan

**Affiliations:** 1 General Surgery, Port of Spain General Hospital, Port of Spain, TTO; 2 Clinical Surgical Sciences, The University of the West Indies, St. Augustine, TTO; 3 Surgery, Medical Associates Hospital, St. Joseph, TTO; 4 Department of Surgery, Eric Williams Medical Sciences Complex, Mount Hope, TTO

**Keywords:** hernia, watchful waiting, severe mental illness, schizophrenia, complication, hernia surgery

## Abstract

Watchful waiting (WW) is a strategy that can be used to manage hernias whereby patients who are asymptomatic or minimally symptomatic are observed until symptoms worsen or complications develop, prompting surgical intervention. The successful implementation of a WW strategy requires patients to report changes in their clinical condition to receive timely care. Patients who have severe mental illness may defer seeking care when appropriate. This case report describes our experience treating a patient with severe mental illness who had a primary ventral hernia managed by WW. She was lost to follow-up and subsequently presented with a strangulated epigastric hernia which fistulized to the skin. The case report highlights the challenges of attempting WW in patients with severe mental illness. We suggest that poorly controlled severe mental illness should be considered a relative contraindication to WW.

## Introduction

Ventral hernias are a common problem, with one in every four individuals developing this condition [1]. Traditionally, surgical repair has been the treatment of choice due to the risks of incarceration and strangulation with their attendant morbidity. However, complications associated with hernia surgery have fueled a desire to identify patients who can be safely managed conservatively. This led to the development of watchful waiting (WW) as a strategy for the management of hernias. During WW, patients who are asymptomatic or minimally symptomatic are observed until symptoms worsen or complications develop, prompting surgical intervention. Both the European and American Hernia societies endorse WW as a safe approach for managing ventral and inguinal hernias [2,3].

The practicality of WW as a management strategy depends on the ability of a patient to present once symptoms worsen. Unfortunately, patients with diminished mental capacity may defer treatment, presenting with advanced complications and the attendant increased risk of morbidity and mortality [4].

Severe mental illness (SMI) is a psychological issue that significantly impairs an individual’s functional and occupational activities. It includes patients with schizophrenia and bipolar disorder [5]. Persons with SMI, when compared to the rest of the population, are more likely to receive suboptimal treatment for physical illness, are less compliant with follow-up and have reduced awareness of physical problems due to reduced cognition and altered pain sensation associated with antipsychotic medication [6]. Consequently, patients with SMI may be less than ideal candidates to have hernias managed by WW.

Here, we present a patient with SMI who had a primary ventral hernia managed by WW. She subsequently presented to our surgical team with a strangulated epigastric hernia which fistulized to the skin. Facilitated by a review of the literature, we make an argument that poorly controlled SMI should be considered a relative contraindication to WW.

## Case presentation

AD is a 72-year-old, morbidly schizophrenic obese female on treatment with risperidone and benztropine. She was seen by another surgical service for an asymptomatic primary ventral hernia. Examination of the abdomen revealed a reducible epigastric hernia with a 3 cm defect. Her hernia was managed with a WW strategy. She defaulted from outpatient surgical follow-up.

**Figure 1:**
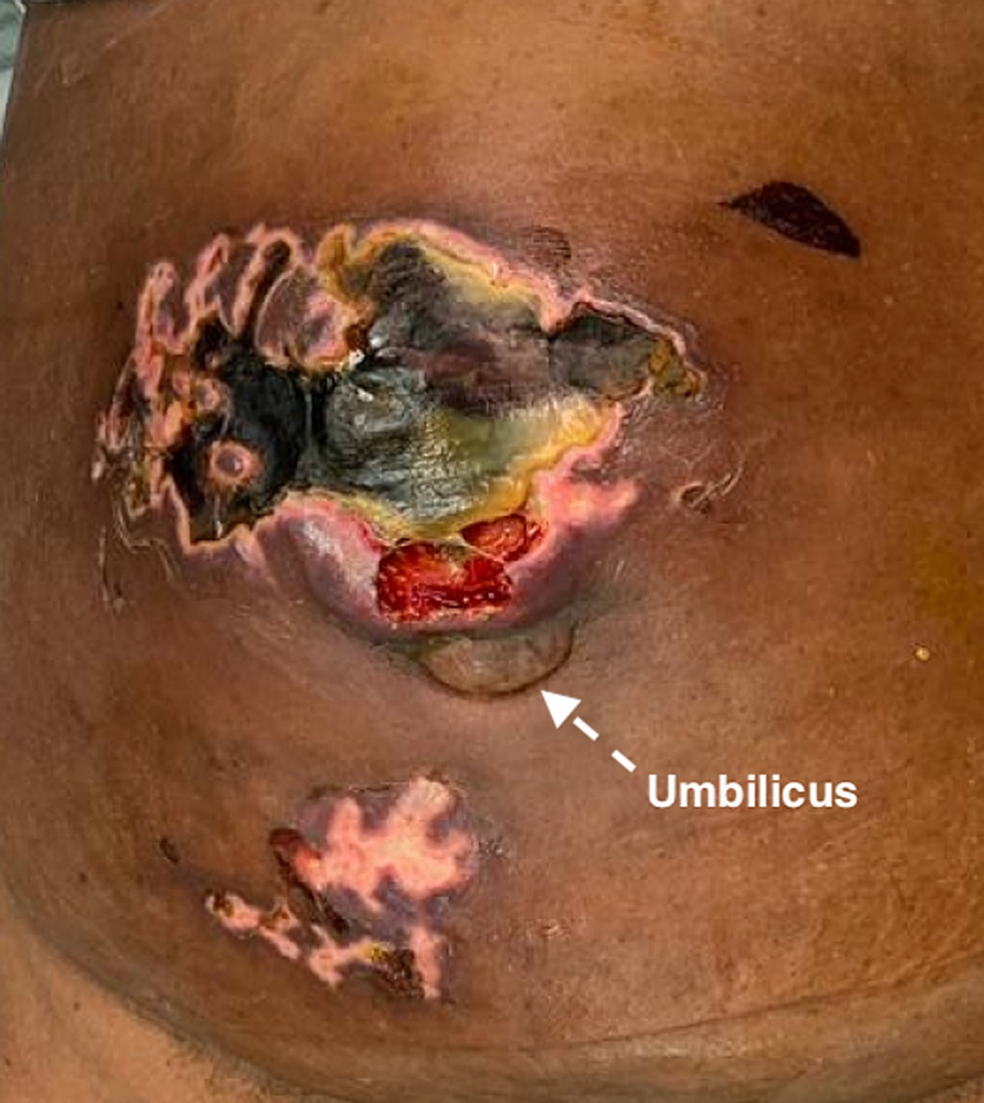
Epigastic hernia with necrotic overlying skin.

**Figure 2:**
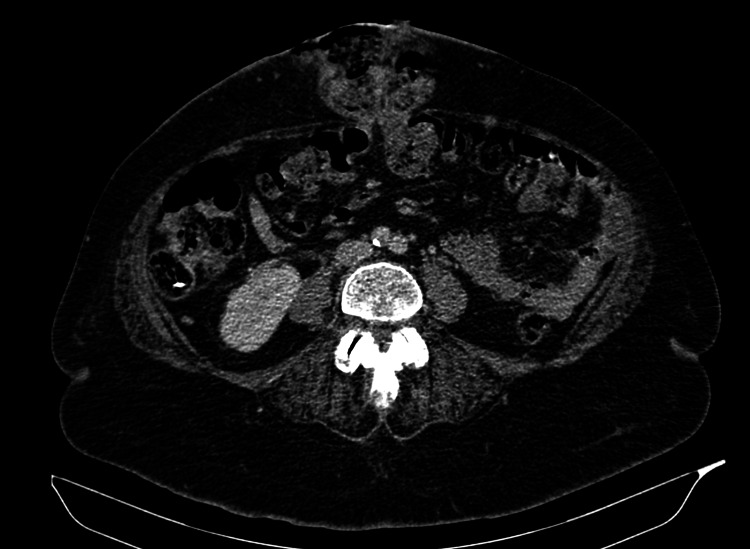
CT of the abdomen showing epigastric hernia containing transverse colon. CT: computed tomography

Eight years later, she presented to the accident and emergency department with a painful abdominal swelling associated with a feculent discharge through the skin. Examination findings were consistent with a strangulated epigastric hernia that sloughed through the overlying skin, discharging stool (Figure 1). A computed tomography scan of the abdomen and pelvis revealed a 3.4 cm epigastric hernia containing transverse colon. There was no evidence of mass lesions, free fluid, or free air in the abdomen (Figure 2).

**Figure 3:**
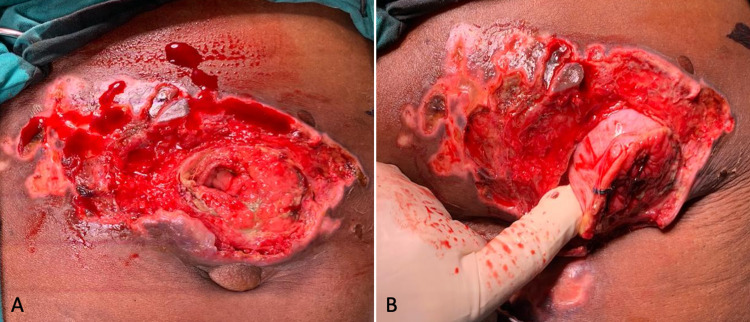
Colocutaneous fistula from transverse colon.

Mental status examination revealed she was not competent to give informed consent. A psychiatrist was consulted before taking the patient for emergency hernia repair. The necrotic skin overlying the hernia was sharply debrided. The hernia sac contained a thickened segment of the transverse colon, which had formed a colocutaneous fistula (Figure 3). The hernia neck was opened, and the transverse colon delivered into the wound. There was no peritoneal soiling. The thickened segment of the colon was resected to healthy bowel, and an end-to-end anastomosis performed. The colon was reduced, and the fascial defect closed with a zero-looped nylon suture. The skin, left open, healed slowly by secondary intention.

She recovered uneventfully, with the return of bowel actions on the second post-operative day. After the resolution of local sepsis, vacuum-assisted dressings were employed. We anticipated poor compliance with outpatient dressings, so her inpatient care extended until the wound epithelialized. She subsequently absconded from follow-up.

## Discussion

The desire to avoid post-operative pain was a motivating factor in developing WW strategies. The incidence of chronic pain after hernia repair is variable, with figures ranging from 0% to 62% for inguinal hernias and from 1% to 20% for ventral hernias [7,8]. Compared to ventral hernias, inguinal hernias occur more frequently and are more likely to be associated with post-operative pain. Unsurprisingly, published literature on WW is mainly focused on inguinal hernias with limited data on ventral hernias.

There are two systematic reviews and one meta-analysis comparing surgery to WW in the treatment of inguinal hernias. The incidence of emergency hernia repair in patients undergoing WW was low in the three studies, ranging from 0.27% to 3%. The low risk of needing emergency surgery led to a common conclusion that WW is safe. However, all three noted a high level of cross-over, with 72% of patients treated with WW requiring surgery during follow-up, with pain being the primary indication for surgery [9-11].

The data on WW for the management of ventral hernias are limited, consisting of small observational studies. WW of ventral hernias is associated with a low risk of requiring emergency surgery (2.6% over one year) [12]. Overall, 16-23% of patients cross over, requiring surgical repair during relatively short follow-up periods of one to five years [13,14]. It has also been demonstrated that patients undergoing ventral hernia repair at index presentation had a statistically significant improvement in their quality of life compared to those who underwent WW [15]. The paucity of evidence translates into weak guideline recommendations in favor of WW for ventral hernias [3,12].

Retrospective data from the United Kingdom have highlighted the potentially deleterious consequences of implementing a WW policy in managing asymptomatic inguinal hernias. Comparison of patients before and after implementation of WW revealed a 59% increase in the risk of emergency hernia repair with an associated increase in adverse events (4.7% vs. 18.5%), including mortality (0.1% vs. 5.4%) [16]. Considering the increased risk of morbidity and mortality associated with emergency hernia repair, surgeons should exercise great caution in counseling patients on WW with a thorough discussion on the potential benefits and risks.

The decision to use a WW strategy should be undertaken on a case by case basis using the best available evidence to guide decision making. Individual patient characteristics can predict their risk of complications and thus their suitability for WW. Data from a registry of 4,472 ventral hernias have revealed that ascites, American Society of Anesthesiologists score III-IV, constipation, increasing body mass index and increasing age are associated with an increased risk of incarceration in primary ventral hernias. Periumbilical and infraumbilical hernias are at increased risk of incarceration compared to other locations. The hernia size also plays a role with defects 3-4 cm in size associated with an increased risk of incarceration compared to smaller (1-2 cm) and larger hernias (≥5 cm). It is thought that in most cases, defects less than 2 cm are too small to permit significant herniation and defects greater than 5 cm are too large to entrap herniated contents [17]. Examining the index case, we can see that the patient had multiple risk factors for incarceration, including advanced age, obesity, periumbilical location, and a 3-4v cm defect size.

The death rate of patients with SMI is 3.7 times higher than that of the general population. They have a higher incidence of medical comorbidities, with an increased risk for cardiovascular and respiratory diseases [5]. The higher prevalence of comorbidities often means patients with SMI are suboptimal surgical candidates. Compared to controls, surgical admissions with SMI have a statistically significant increased rate of post-operative complications (22% vs. 8%), longer hospital stays (12.2 days vs. 4.6 days), higher admission costs ($24,162 vs. $12,336), and higher in-hospital mortality (2% vs. 0%) [18]. The increased incidence of emergency surgical care versus elective and poorer access to treatment contributes to the worse outcomes observed in these patients [19]. This data is reflected in our case, which needed emergency surgery, had a prolonged hospital course and was lost to follow-up.

There are no published articles on WW as a management strategy in patients with known SMI. However, given their predisposition to complications, surgeons should be cautious in using WW for patients with SMI. Emergency hernia repairs have a higher risk of morbidity and mortality when compared to elective cases and should thus be avoided where possible in high-risk groups [4]. Furthermore, individuals managed with WW play an essential role in their care, alerting surgeons to changes in their clinical status, indicating the need for surgical intervention. Unfortunately, patients with SMI may lack the capacity to recognize significant changes and, consequently, present with complicated hernias necessitating emergency surgery, as seen in the index case.

Until further research becomes available to clarify the role of WW in patients with SMI, it is our opinion that SMI should be considered a relative contraindication to WW. Persons with SMI who are poor surgical candidates due to uncontrolled comorbidities or limited life expectancy may still be considered for WW, but this should be done using a multidisciplinary approach. Engaging the assistance of psychiatrists, psychologists, patient advocates, and social workers can aid in compliance with clinical follow-up. Follow-up is key as it allows for timely identification of changes in hernia status to facilitate prompt surgical intervention and avoid emergency repair.

Surgeons are more likely to overlook comorbid psychiatric disorders than physical comorbidities. They infrequently ask about patients’ mental health and report low confidence in caring for patients with mental health issues [20]. Educational initiatives must be undertaken to enlighten surgeons on the unique needs of this disadvantaged population to improve surgical outcomes. A multidisciplinary approach to their management should be routine, with the inclusion of psychiatrists as part of an integrated surgical care team to guide the management of patients with SMI.

## Conclusions

WW is a management strategy that can be used to treat asymptomatic and minimally symptomatic hernias. While WW is reported to be safe, it may increase the risk of emergency hernia repair, which is associated with increased morbidity and mortality. Patients with SMI are known to have decreased compliance with medical management and poorer surgical outcomes. Reduction in the capacity of patients with SMI may increase the risk of emergency hernia repair in patients undergoing WW. Until further research is available, SMI should be considered a relative contraindication to WW.

## References

[1] Bedewi MA, El-Sharkawy MS, Al Boukai AA, Al-Nakshabandi N (2012). Prevalence of adult paraumbilical hernia. Assessment by high-resolution sonography: a hospital-based study. Hernia.

[2] HerniaSurge Group (2018). International guidelines for groin hernia management. Hernia.

[3] Henriksen NA, Montgomery A, Kaufmann R (2020). Guidelines for treatment of umbilical and epigastric hernias from the European Hernia Society and Americas Hernia Society. Br J Surg.

[4] Lebeau R, Traoré M, Anzoua KI (2016). Prognostic factors of postoperative morbidity and mortality of adult strangulated groin hernia. Indian J Surg.

[5] (2021). Severe mental illness (SMI) and physical health inequalities: briefing - GOV.UK. https://www.gov.uk/government/publications/severe-mental-illness-smi-physical-health-inequalities/severe-mental-illness-and-physical-health-inequalities-briefing.

[6] De Hert M, Cohen D, Bobes J (2011). Physical illness in patients with severe mental disorders. II. Barriers to care, monitoring and treatment guidelines, plus recommendations at the system and individual level. World Psychiatry.

[7] Helgstrand F (2016). National results after ventral hernia repair. Dan Med J.

[8] Hakeem A, Shanmugam V (2011). Inguinodynia following Lichtenstein tension-free hernia repair: a review. World J Gastroenterol.

[9] Mizrahi H, Parker MC (2012). Management of asymptomatic inguinal hernia: a systematic review of the evidence. Arch Surg.

[10] Gong W, Li J (2018). Operation versus watchful waiting in asymptomatic or minimally symptomatic inguinal hernias: the meta-analysis results of randomized controlled trials. Int J Surg.

[11] Reistrup H, Fonnes S, Rosenberg J (2020). Watchful waiting vs repair for asymptomatic or minimally symptomatic inguinal hernia in men: a systematic review. Hernia.

[12] Liang MK, Holihan JL, Itani K (2017). Ventral hernia management: expert consensus guided by systematic review. Ann Surg.

[13] Holihan JL, Flores-Gonzalez JR, Mo J, Ko TC, Kao LS, Liang MK (2017). A prospective assessment of clinical and patient-reported outcomes of initial non-operative management of ventral hernias. World J Surg.

[14] Kokotovic D, Sjølander H, Gögenur I, Helgstrand F (2016). Watchful waiting as a treatment strategy for patients with a ventral hernia appears to be safe. Hernia.

[15] Bernardi K, Martin AC, Holihan JL (2019). Is non-operative management warranted in ventral hernia patients with comorbidities? A case-matched, prospective 3 year follow-up, patient-centered study. Am J Surg.

[16] Hwang MJ, Bhangu A, Webster CE, Bowley DM, Gannon MX, Karandikar SS (2014). Unintended consequences of policy change to watchful waiting for asymptomatic inguinal hernias. Ann R Coll Surg Engl.

[17] Sneiders D, Yurtkap Y, Kroese LF, Kleinrensink GJ, Lange JF, Gillion JF (2019). Risk factors for incarceration in patients with primary abdominal wall and incisional hernias: a prospective study in 4472 patients. World J Surg.

[18] McBride KE, Solomon MJ, Young JM, Steffens D, Lambert TJ, Glozier N, Bannon PG (2018). Impact of serious mental illness on surgical patient outcomes. ANZ J Surg.

[19] Reeves E, Henshall C, Hutchinson M, Jackson D (2018). Safety of service users with severe mental illness receiving inpatient care on medical and surgical wards: a systematic review. Int J Ment Health Nurs.

[20] McBride KE, Solomon MJ, Lambert T, O'Shannassy S, Yates C, Isbester J, Glozier N (2021). Surgical experience for patients with serious mental illness: a qualitative study. BMC Psychiatry.

